# Culture-free Antibiotic-susceptibility Determination From Single-bacterium Raman Spectra

**DOI:** 10.1038/s41598-018-22392-9

**Published:** 2018-03-02

**Authors:** A. Novelli-Rousseau, I. Espagnon, D. Filiputti, O. Gal, A. Douet, F. Mallard, Q. Josso

**Affiliations:** 1 bioMérieux, Technological Research Department, 5 rue des Berges, 38024 Grenoble, France; 2 CEA, LIST, Département Métrologie, Instrumentation et Information, 91191 Gif-sur-Yvette, France; 3 Bioaster, 40 avenue Tony Garnier, 69007 Lyon, France

## Abstract

Raman spectrometry appears to be an opportunity to perform rapid tests in microbiological diagnostics as it provides phenotype-related information from single bacterial cells thus holding the promise of direct analysis of clinical specimens without any time-consuming growth phase. Here, we demonstrate the feasibility of a rapid antibiotic-susceptibility determination based on the use of Raman spectra acquired on single bacterial cells. After a two-hour preculture step, one susceptible and two resistant *E*. *coli* strains were incubated, for only two hours, in the presence of different bactericidal antibiotics (gentamicin, ciprofloxacin, amoxicillin) in a range of concentrations that included the clinical breakpoints used as references in microbial diagnostic. Spectra were acquired and processed to isolate spectral modifications associated with the antibiotic effect. We evidenced an “antibiotic effect signature” which is expressed with specific Raman peaks and the coexistence of three spectral populations in the presence of antibiotic. We devised an algorithm and a test procedure that overcome single-cell heterogeneities to estimate the MIC and determinate the susceptibility phenotype of the tested bacteria using only a few single-cell spectra in four hours only if including the preculture step.

## Introduction

Today, the initial antibiotherapies are usually empiric and suboptimal since antibiotic susceptibility data are not available on the collection day of the sample. Consequently, developing new rapid microbiological diagnosis and more specifically Antibiotic Susceptibility Testing (AST) that would give a response in a few hours instead of a few days is a key challenge not only to promote optimal patient outcomes but also to attenuate or stop the development of new antibiotic-resistant organisms^1,2^. Standard methods routinely used for AST require several preparatory steps such as growth and isolation on solid agar culture medium for 18 to 24 hours before assays such as the gold standard broth microdilution assay can be performed. Typically, the test results in the determination of the Minimum Inhibitory Concentration (MIC) of the considered strain for the given antibiotic. The comparison of this MIC to reference breakpoints defined by institutions such as the Clinical and Laboratory Standards Institute (CLSI, http://clsi.org)^3^ and the European Committee on Antimicrobial Susceptibility Testing (EUCAST, http://www.eucast.org)^4^ allows to qualify a strain as resistant (R), intermediate (I) or susceptible (S) to the antibiotic. Even in automated formats such as Vitek-2 (bioMérieux, Marcy l’Etoile, France) or Phoenix (Becton Dickinson, Sparks, MD, USA), AST from bacterial colonies still takes 6–10 hours after isolation of colonies, leading to actionable results being delivered 30 to 72 hours after sampling. As a consequence, test results are not used to tailor the initial therapeutic decision and to reduce broad spectrum antibiotic usage. MALDI-TOF has helped to speed up the microbial identification – although it still requires bacterial growth – and it is now key to speed up the AST^5^ in order to provide actionable information sooner to clinicians. Nowadays, one of the fastest approaches for resistance detection consists in molecular biology – mostly PCR-based – assays. Such assays only require small numbers of micro-organisms and can be performed directly from a clinical specimen in just hours without requiring any time-consuming bacterial culture. Nevertheless, such tests are based on amplification of known antibiotic resistance genes and inherently fail to detect unknown or unexpected phenotypes^6,7^. Alternatively, several phenotypic approaches, based on optical methods, enable direct analysis of single microorganisms present in a clinical sample. Fluorescence microscopy and phase-contrast imaging have recently been used to test the direct effect of antibiotics on cell morphology and early division cycles^8,9^. Although they may reach single-cell sensitivity and provide excellent performance in some cases, such methods are still limited by the growth speed and structural change characteristic of the microbial species. Probing the initial metabolic response of single micro-organisms to the presence of antibiotics, independently of any morphological changes and division, would further accelerate testing. Among optical methods adapted to the characterization of individual cells, Raman micro-spectroscopy has been shown to accurately provide identification information from single bacterial cells^10–14^ and has also been shown to detect metabolic changes at the single bacterial cell level when they are incubated in presence of antibiotics^15–17^. More advanced methods using Surface Enhanced Raman Spectroscopy have also been demonstrated^18^ that could provide several advantages regarding the acquisition time^19^ or the selectivity^20^ (if required) at the cost of some additional reagents or protocol complexity. However, most of the related studies were restricted to very high antibiotic concentrations^15^. Other studies have demonstrated that resistance phenotypes can be detected with antibiotic concentrations in the scope of AST but when averaging the response of a number of cells^21–24^. Demonstrating that Raman microspectroscopy is able to perform single-cell AST tests would provide a technique that is both able to provide identification and AST. It would be a major improvement of the overall achievable time to phenotypic results. In the present study, we performed Raman micro-spectrometry at the level of single bacterial cells incubated in the presence of antibiotic concentrations close to clinical breakpoints. We observed that in such conditions, Raman spectra are modified in a unique manner in the presence of antibiotic, defining what could be called an “antibiotic effect signature”. We then studied the occurrence of this “signature” inside a population of bacterial cells as a function of the antibiotic concentration. Interestingly, we show that the antibiotic effect signature is unequally spread among the bacterial cells population and that this variability reflects the coexistence of different spectral populations which relative abundance varies depending on antibiotic concentration. Furthermore, we demonstrate, by analyzing these differences through a Support Vector Machine (SVM) with a radial basis kernel, that this differential spectral behavior can be used to determinate the susceptibility phenotype and estimate the MIC of the considered strain, and that this prediction can be done using only a very small number of cells of the considered bacterial population.

## Results

### Study design

The standard broth microdilution test used to determine the MIC of an antibiotic relies on a series of overnight bacterial cultures in presence of the antibiotic at increasing concentrations. The concentration range used corresponds to a series of doubling concentrations including the low and high breakpoints as they are defined by the CLSI and by the EUCAST (Table 1 and Methods) and depends on the bacterial species. The monitoring of the growth in each culture allows the determination of the MIC for which bacteria do not grow. MIC under the low breakpoint defines susceptible strains while MIC values above the high breakpoint define resistant strains.

**Table 1:** Description of the biological model and experimental database. Three antibiotics have been tested: gentamicin which inhibits protein synthesis, ciprofloxacin which prevents DNA supercoiling, and amoxicillin which impedes the cell wall synthesis. For each of them are listed the clinical breakpoints from EUCAST^4^ for *E*. *coli*, the tested concentrations, the references of susceptible and resistant strains of *E*. *coli*, with the corresponding MICs (measured in Vitek®2). Each “experiment” consisted in testing, on one date, one strain in presence of one antibiotic at multiple concentrations (including 0) and acquiring around 50 spectra per concentration; it was replicated on different dates. The overall database contains 3668 spectra of single bacteria. Note that *E*. *coli* ATCC 25922 strain is the AST quality-control reference recommended by EUCAST.

Antibiotic	Gentamicin	Ciprofloxacin	Amoxicillin
MIC breakpoints for *E*. *coli* (µg/mL)			
Susceptible	S ≤ 2	S ≤ 0.25	S ≤ 8
Resistant	R > 4	R > 0.5	R > 8
Tested concentrations (µg/mL)	0.5, 2, 8 (128, 256)*	0.005, 0.015, 0.064, 0.25, 1	2, 4, 8, 16
Susceptible strain	ATCC 25922 (EC1)	ATCC 25922 (EC1)	ATCC 25922 (EC1)
MIC (µg/mL)	1	0.008	6
Number of experiments/spectra	7/886	7/889	5/604
Resistant strain	ATCC 35421 (EC2)	API 9210041 (EC3)	ATCC 35421 (EC2)
MIC (µg/mL)	>256	3	>256
Number of experiments/spectra	3/404	3/399	3/486

^*^Only tested on resistant strain EC2.

Here, based on the same principle, we incubated a series of bacterial suspensions in the presence of antibiotics at increasing concentrations ranging from below low breakpoint to above high breakpoint defined by the EUCAST (see Table 1). Bacteria cultures without antibiotic were also incubated as a reference. The incubation time in presence of antibiotics was reduced to two hours before performing a Raman chemometric analysis of single cells. For that purpose, bacteria were collected from the suspension and deposited onto a glass coverslip for Raman spectra acquisition on single bacterial cells. After spectra acquisition, each spectrum was preprocessed to remove the background signal (mostly fluorescence) and glass signal (see details in Methods) in order to reduce the impact of non-biological and non-specific signal^25^. The resulting spectra, normalized by their mean intensity in the region of interest [650–1750] cm^−1^, were called “normalized net spectra”. For the sake of clarity, in the following description, bacteria which were incubated for 2 h with antibiotic are called “exposed” bacteria and bacteria which were incubated without antibiotic “non-exposed” bacteria. The test was performed both on a susceptible strain and on resistant strains for each antibiotic molecule (see Table 1) in order to confirm the specificity of the detected effect. The method was carried out for three bactericidal antibiotics with different modes of action, an aminoglycoside, a quinolone and a *beta*-lactam. Resulting experimental conditions are summarized in Table 1. For each tested antibiotic, viability assays which consisted in culturing the samples on agar culture medium directly after incubation with antibiotic were performed. It allowed us to confirm that in our conditions the antibiotics were effective on bacterial cells and led to a loss of viability as could be expected for the different antibiotic concentrations (see supplementary Fig. 1). No loss of viability was observed for resistant strains. We confirmed that the number of cells was not smaller after than before the sample incubation by counting cells using microscopy (data not shown). This numbering, coupled to a more classical Petri dish numbering, confirmed that we were able to observe, and gather spectra from, bacteria that were still in the sample but no longer able to grow. We hence ascertained that no bias was introduced in our study by selecting a specific population of cells due to the sample preparation protocol and that we were measuring effects on cells that were undergoing an effect of the antibiotic.

### Extraction of the antibiotic-effect spectral signature

**Figure 1:**
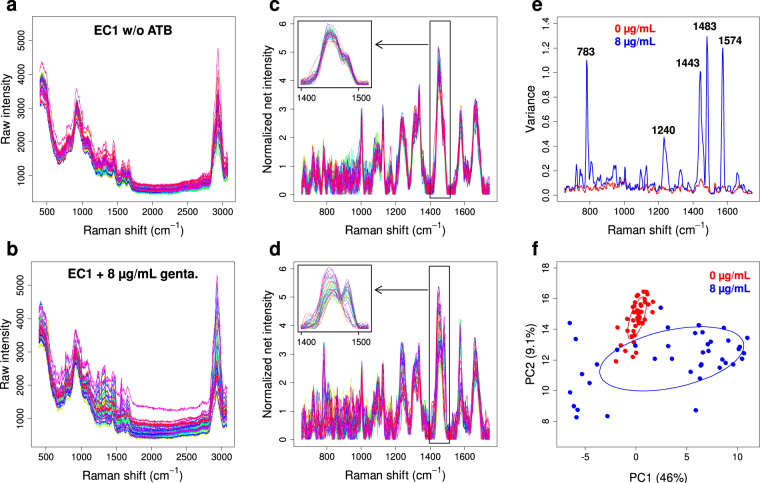
Raman spectra of single bacterial cells from susceptible *E*. *coli* strain EC1 incubated for 2 hours in the absence or presence of 8 µg/mL gentamicin. Raw spectra in the (**a**) absence (44 spectra) and (**b**) presence (39 spectra) of antibiotic, obtained in a single experiment; (**c**,**d**) corresponding normalized net spectra in the region of interest [650–1750] cm^−1^ with a zoom on region [1400–1520] cm^−1^, and (**e**) associated variance spectra of the bacteria incubated with (blue) and without (red) antibiotic; (**f**) PCA plot of the normalized net spectra, evidencing the enhanced variance among exposed bacteria (blue dots) and their discrimination from non-exposed bacteria (red dots). The corresponding 50%-confidence ellipses are plotted as a visual guide.

In order to extract the Raman signature of the antibiotic effect on single bacteria, we first analyzed the changes induced on Raman spectra of single bacterial cells when they were exposed to inhibitory antibiotic concentrations. Such modifications are illustrated by Fig. 1 in the case of susceptible *E*. *coli* cells (EC1; see Table 1) incubated for 2 hours in the absence or presence of gentamicin at eight times the related MIC (8 µg/mL). Although spectral changes between exposed and non-exposed bacteria were very faint (see raw spectra in Fig. 1a,b and normalized net spectra in Fig. 1c,d), they could clearly be evidenced on a small number of Raman peaks (see zoom in Fig. 1c,d). They were accompanied with a much higher intercellular spectral variability in the presence of antibiotic, concentrated on few specific Raman peaks (Fig. 1e). The principal component analysis (PCA) of normalized net spectra (Fig. 1f) clearly showed this enhanced variability among spectra of exposed bacteria, as also a partial discrimination from spectra of non-exposed bacteria. This suggested that the antibiotic effect was not homogeneous among individual cells in the bacterial population, although the loss of viability in those conditions was more than 99.99% (see supplementary Fig. 1). Very similar spectral modifications could be observed on the same strain in the presence of inhibiting concentrations of ciprofloxacin and amoxicillin (not shown).

In order to isolate the spectral changes that were specific to the antibiotic effect and that occurred with small amplitude, we calculated “difference spectra” defined as the difference between each normalized net spectrum and the mean of all the normalized net spectra measured on the non-exposed bacterial cells in the same experiment (i.e. same strain and date). This procedure was designed to remove the main part of the strain-specific information and the metabolic variability originating from the bacteria at different dates and evidences spectral changes that are reproducible for different dates and strains.

**Figure 2:**
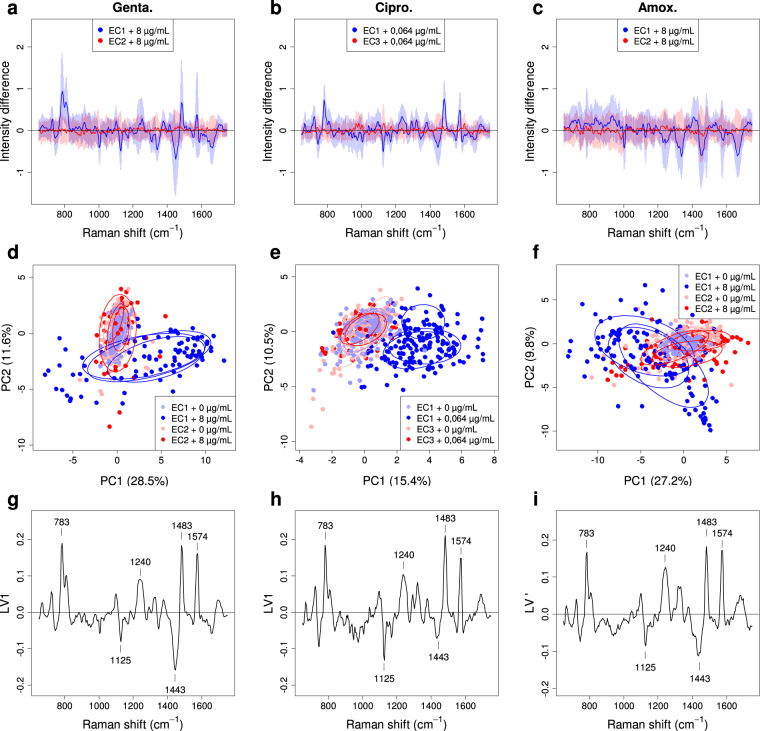
Spectral signature of the antibiotic effect extracted from the difference spectra of single bacterial cells. The “difference spectra” (*i*.*e*. the differences between each normalized net spectrum and the mean of all the normalized net spectra measured on the non-exposed bacterial cells) of single bacterial cells incubated for 2 hours are shown for (**a**) gentamicin (0 and 8 µg/mL, 374 spectra), (**b**) ciprofloxacin (0 and 0.064 µg/mL, 483 spectra) and (**c**) amoxicillin (0 and 8 µg/mL, 474 spectra). For clarity, only the mean of the difference spectra is shown, for the exposed susceptible bacteria (blue line) and the exposed resistant ones (red line), as well as the corresponding ±σ standard-deviation range (light blue and pink bands). (**d–f**) Corresponding PCA plots of difference spectra, for susceptible bacteria exposed (dark-blue dots) and non-exposed (light-blue dots) as well as for resistant bacteria exposed and non-exposed (resp. red and pink dots). 50%-confidence ellipses are also plotted with the same colors, independently for each of the three experiments dates, as a visual guide to show the reproducibility of experiments. (**g–i**) Plots of the loading vectors, LV1 for (**g**) gentamicin and (**h**) ciprofloxacin, giving the weights involved in the PC1 score, and LV’ = (LV1–LV2) × √2/2 for (**i**) amoxicillin, giving the weights involved in the (PC1–PC2) × √2/2 score. These loading vectors constitute the “spectral signature” of the antibiotic effect.

By doing so, we could clearly detect the antibiotic effect on the susceptible strain. The results, presented in Fig. 2 for the three antibiotics, showed that the difference spectra from the exposed susceptible bacterial cells exhibited significant and reproducible positive and negative peaks. This is illustrated through the mean of those difference spectra, plotted as a blue line in Fig. 2a–c and accompanied with the aforementioned high intercellular variability, concentrated on the same peak positions (±*σ* range around the mean shown as a light-blue band in Fig. 2a–c). The peaks were more obvious for gentamicin (Fig. 2a) and ciprofloxacin (Fig. 2b) than for amoxicillin (Fig. 2c), where they were considerably reduced by the averaging. In contrast, no significant spectral modification was observed for the exposed resistant bacteria (mean of their difference spectra is plotted as a red line in Fig. 2a–c) for which the cell-to-cell variability was also found to be lower (pink band). This confirms the specificity of the spectral changes induced by the antibiotic on susceptible strain. For the three antibiotics, PCA plots of same difference spectra (Fig. 2d–f) showed more obvious spectral changes – including for amoxicillin – which significantly separated the exposed (dark-blue dots) from the non-exposed (light-blue dots) susceptible bacterial cells, the latter being superimposed precisely on the exposed (red dots) and non-exposed (pink dots) resistant bacteria. The PCA plots also showed that the results for the exposed susceptible bacteria were quite reproducible from date to date (dark-blue ellipses superimposed for the 3 experiment dates in Fig. 2d–f).

**Figure 3:**
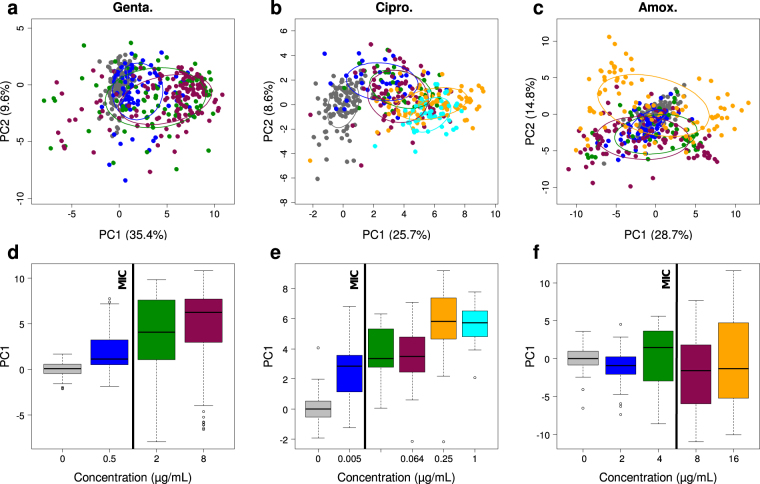
Analysis of the dose-effect relationship for the three antibiotics. (**a–c**) PCA plots of the difference spectra (previously defined in text and in Fig. 2) for single bacterial cells of susceptible strain (EC1) incubated for 2 hours with (**a**) gentamicin (382 spectra), (**b**) ciprofloxacin (345 spectra) and (**c**) amoxicillin (418 spectra) at different concentrations (two to four independent experiments per concentration and antibiotic in most cases), with 50%-confidence ellipses plotted for each concentration. (**d**–**f**) Box plots of the distributions of the PC1 scores for the different antibiotic concentrations (indicating the correspondence between colors and concentrations). Vertical black lines show the reference MICs.

**Figure 4:**
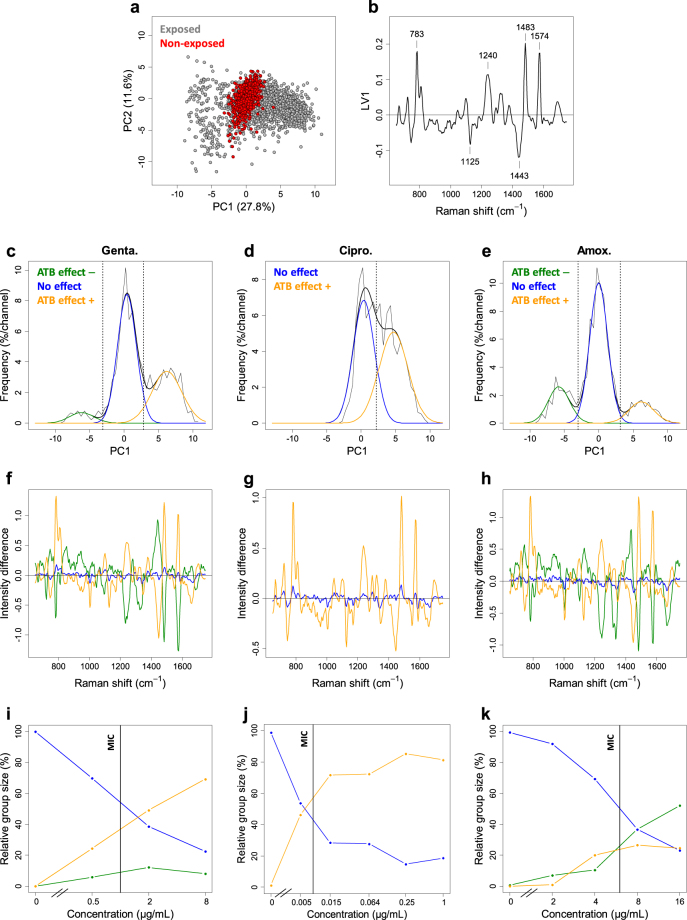
Analysis of the heterogeneity of spectral modifications for the susceptible bacterial strain EC1 exposed to different antibiotics. (**a**) Global PCA of the difference spectra (2481 spectra) of single bacterial cells from the susceptible strain EC1 for the three antibiotics at all concentrations. The dots corresponding to exposed (resp. non-exposed) cells are plotted in gray (resp. red), evidencing an antibiotic-effect discrimination parallel to the PC1 axis. (**b**) Corresponding LV1 loading vector considered as the common spectral signature of the antibiotic effect. (**c**–**e**) Separated histograms of the PC1 scores of the difference spectra for each antibiotic. The histograms clearly show two [for ciprofloxacin (**d**)] or three [for gentamicin (**c**) and amoxicillin (**e**)] distribution peaks. The corresponding populations are delimited by fitting the histograms with (2 or 3) Gaussian distributions and placing score thresholds at values that equalize tail areas of overlapping distributions [vertical dotted lines in (**c**–**e**)]. The color code used for the Gaussian fits is common to (**c**–**k**) and explained hereafter. Each difference spectrum has thus been attributed to one of the three groups according to its PC1 score. The resulting mean difference spectra of the three groups are shown in (**f**–**h**). The group of difference spectra with low scores around 0 contains the difference spectra of all non-exposed bacteria and is called the “No effect” group (blue distribution). The group of spectra with high positive scores (called “ATB effect + ”, orange distribution) and the group of spectra with high negative scores (called “ATB effect −”, green distribution) are specific of exposed, susceptible bacteria. (**i**–**k**) Plots of the relative sizes of the 3 groups against antibiotic concentration for each antibiotic. For ciprofloxacin (**d**,**g**,**j**), the “ATB effect −” group is not observed. The vertical black lines in (**i**–**k**) show the reference MIC values.

In the PCA plots, the main separation between the spectra from exposed susceptible bacteria and those from non-exposed or resistant bacteria was observed parallel to the axis of the first “principal component” (PC1) for gentamicin and ciprofloxacin (Fig. 2d,e). For amoxicillin (Fig. 2f), and for this particular restricted set of data, it was rather parallel to the PC1–PC2 direction. (For larger amoxicillin datasets, incorporating more concentrations, as shown in Fig. 3 or Fig. 4, the PC1 axis was more closely aligned with the observed shifts.) That is why we examined the “loading vector” LV1 (Fig. 2g,h for gentamicin and ciprofloxacin) or LV’ = (LV1–LV2) × √2/2 (Fig. 2i for amoxicillin), which gives the spectral features that contribute to the PC1 score, or respectively to the (PC1–PC2) × √2/2 score, with their weights (positive or negative). A positive score corresponds to an increase in peaks with positive weight in the loading vector and a decrease in peaks with negative weight, compared to spectra of non-exposed bacterial cells (and the contrary for a negative score). This vector thus constituted a pertinent “spectral signature” for the antibiotic effect. Strikingly, the signatures obtained for the three antibiotics were highly similar (Fig. 2g–i). It is also noticeable that, for gentamicin (Fig. 2d) and amoxicillin (Fig. 2f), the antibiotic effect was reflected both by spectra with a positive shift and by spectra with a negative shift in PC1 (resp. PC1–PC2). This was not the case with ciprofloxacin (Fig. 2e), for which only positive shifts were observed. This will be further discussed in the subsequent sections. The spectral zones significantly contributing to the signature correspond to spectral features typical of bacterial Raman spectra: peaks with positive weights at 783 cm^1^, 1240 cm^−1^, 1483 cm^−1^ and 1574 cm^−1^, are attributed to nucleic acid^15^ whereas peaks at 1125 cm^−1^ and 1443 cm^−1^, with negative weights, are generally attributed to proteins^15^.

### Analysis of the dose-effect relationship

In order to determine whether this spectral signature of the antibiotic effect and the associated score could be directly used to determine the MIC of the susceptible strain, we studied the variations (and distributions) of the PC1 score of single EC1 bacterial cells incubated with increasing concentrations of antibiotic, from well under (including 0) to well above the MIC. The results obtained for the three antibiotics (see Fig. 3) showed that the PC1 score tended to increase with increasing antibiotic concentration, at least for gentamicin and ciprofloxacin (Fig. 3d,e). However, this variation was largely masked by the high cell-to-cell spectral variability (Fig. 3a–c) that we observed, especially at high antibiotic concentrations. In the case of amoxicillin, decreases of PC1 score with increasing concentration could even be observed (Fig. 3f, between 0 and 2 µg/mL, and between 4 and 8 µg/mL). Note that, in these new PCA plots, the LV1 loading vector associated with PC1 (not shown) was very similar to the spectral signature shown in Fig. 2g–i.

To further analyze the distributions of the PC1 scores for different concentrations of the antibiotic, while taking into account the fact that very similar spectral signatures had been found for the antibiotic effect with the three antibiotics, we calculated a global PCA for all difference spectra measured on the single bacterial cells of the susceptible strain (all three antibiotics, all concentrations, all experiments). The main discriminating axis for the antibiotic effect was found parallel to PC1 again, with about no discrimination along the PC2 axis (see Fig. 4a). The loading vector LV1 associated with the principal component PC1 was then taken as the common spectral signature of the antibiotic effect (Fig. 4b) and we plotted the global distribution of the PC1 scores, for each antibiotic (Fig. 4c–e). The resulting histograms clearly showed a bimodal (for ciprofloxacin) or trimodal (for gentamicin and amoxicillin) repartition of spectra, suggesting the existence of distinct populations of bacterial conditions. These populations were delimited by fitting the histograms with (two or three) Gaussian peaks. A first population with low scores (around zero) corresponding to flat difference spectra (mean difference spectrum shown in blue in Fig. 4f–h) was called “no effect” group. A second population, with high positive scores, was called “ATB effect + ” group. Its mean difference spectrum (shown in orange in Fig. 4f–h) is characterized by positive peaks at 783 cm^−1^, 1240 cm^−1^, 1483 cm^−1^ and 1574 cm^−1^ and negative peaks at 1125 cm^−1^ and 1443 cm^−1^. The third population, with high negative scores, was called “ATB effect −” group. This last group has a mean difference spectrum with about the same peaks and opposite intensities (in green in Fig. 4f,h; not observed for ciprofloxacin). To further characterize the relationship between antibiotic concentration and spectral effect, we calculated the relative abundance of each of these three groups among the difference spectra, as a function of antibiotic concentration. The resulting plots (Fig. 4i–k) showed that the “no effect” group contained all non-exposed bacteria, as expected. When increasing the antibiotic concentration, the relative size of this group gradually decreased. On the contrary, “ATB effect+” and “ATB effect−” groups were observed only among the exposed bacteria (except for ciprofloxacin for which “ATB effect−” group was not observed) and gradually increased their size with increasing antibiotic concentration. This important observation shows that, at the single bacterial cell level, the modification of Raman spectral properties (hence metabolic state) does not change in a continuous manner. Instead, bacterial cells show or do not show the antibiotic effect signature in their spectrum and only the proportion of bacterial cells with modified spectrum changes with antibiotic concentration. This population-based model is a key element to fully capture and understand the effect of antibiotics through Raman spectra measurements.

Note that the coexistence of these two or three groups at a given antibiotic concentration, with possibly comparable relative sizes, largely explains the high variance observed among spectra of exposed susceptible bacteria described above. This also explains why this variance is mainly concentrated on the 6 peak positions listed above. In addition, we noticed that, for each antibiotic, the “no effect” group decreased below 50% while the two other groups became a majority at concentrations around the MIC (see MIC values in Table 1), suggesting that the observation of single cell Raman spectra could be used to estimate the MIC of a given strain. As described in the next paragraphs, we confirmed that opportunity by developing a direct method for the MIC determination based on machine learning and using only a few spectra of single bacteria for each concentration.

### Automatic antibiotic-effect detection and MIC determination

The basis for our approach was to use supervised machine learning to automatically recognize the antibiotic effect in a few single bacterial cell spectra and, by repeating this test for a series of bacterial suspensions of the same strain incubated with increasing concentrations of antibiotic, to detect whether a transition occurred and at which concentration compared to the reference MIC. The first step was, for each antibiotic, to build a learning database of difference spectra belonging to two classes: a “No effect” class, with the difference spectra from non-exposed susceptible bacterial cells, and an “ATB effect” class with only the difference spectra from the susceptible bacterial cells exposed to inhibitory concentrations of antibiotic (≥2 µg/mL for gentamicin, ≥0.005 µg/mL for ciprofloxacin and ≥8 µg/mL for amoxicillin). All spectra from the resistant strains were excluded from this learning database.

For classification, the SVM algorithm was used with a “radial basis function” (RBF) kernel^26^ (software available at http://www.csie.ntu.edu.tw/~cjlin/libsvm) to deal with the non-linear nature of the observed effect, especially the coexistence of the opposite “ATB effect+” and “ATB effect−” populations among the difference spectra of the exposed susceptible bacteria. Note that, although we used the full difference spectra for the classification, we observed that a linear SVM had a limited performance for this task. This suggests that the full data were not or poorly linearly separable due to the aforementioned opposite populations, as evidenced in two dimensions by the PCA (Fig. 4a), hence the use of a RBF kernel. To ensure a robust and stringent validation of the classifier, avoiding any overfitting artefact or confounding effect, we followed a “leave-one-date-out” cross-validation scheme. For that purpose, we built the validation partitioning by grouping all difference spectra corresponding to a given date together in the same test group. That group was excluded from the learning reference when tested. Of course, this was realized by construction in the case of the resistant strains or lower concentrations since they were not included in the learning database. With this validation scheme, the spectra of each experiment were tested by only using spectra from other experiments as reference.

**Figure 5:**
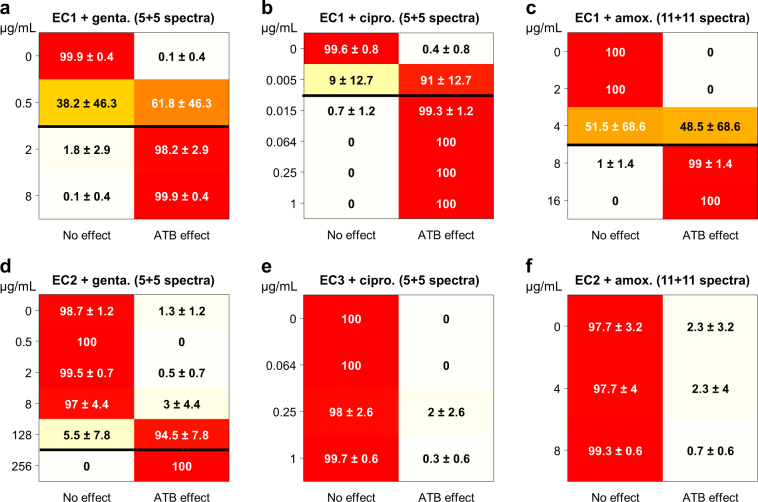
Confusion matrices for automatic discrimination between classes “No effect” and “ATB effect” at different antibiotic concentrations by a SVM classifier. The matrices are plotted for susceptible strain EC1 and (**a**) gentamicin (847 spectra), (**b**) ciprofloxacin (850 spectra) and (**c**) amoxicillin (602 spectra), and for resistant strains (**d**) EC2 and gentamicin (404 spectra), (**e**) EC3 and ciprofloxacin (399 spectra), and (**f**) EC2 and amoxicillin (366 spectra). For each antibiotic concentration (one row of the matrix), the figures report the classification rate in each of the “No effect” and “ATB effect” classes (in %, with its standard deviation calculated among the 2 to 7 independent experiments at the considered concentration). Horizontal black lines show reference MIC values for the susceptible strain (**a**–**c**), as well as the observed complete transition observed for resistant strain EC2 and gentamicin (**d**) when extending the concentration range to very high values.

Due to the high intercellular spectral variability, including the presence of exposed bacteria without observable spectral changes, it was necessary to repeat the test on a small number of difference spectra to reliably decide whether the bacterial cells were susceptible or not to the antibiotic at a given concentration. In view of validating the test in realistic conditions, as close as possible to a direct sample clinical application, we restricted the mean spectrum of non-exposed bacteria used in the calculation of the difference spectra to the same small number of non-exposed bacteria. We could reach satisfying performance with only 5 difference spectra for gentamicin and ciprofloxacin (from 5 exposed and 5 non-exposed cells) and 11 difference spectra for amoxicillin (from 11 exposed and 11 non-exposed cells). Each of the 5 (or 11) tested difference spectra was labeled by the classifier and a majority vote was applied to assign a class to the tested set. The detailed performances are represented as confusion matrices in Fig. 5 (see also supplementary Fig. 2–4 for confusion matrices obtained with other numbers of difference spectra). For the susceptible strain (Fig. 5a–c), a sharp transition was observed for concentrations around the MIC, from a “No effect” labelling under the MIC to an “ATB effect” labelling above the MIC. For the resistant strains (Fig. 5d–f), no antibiotic effect was detected by the classifier, regardless of the antibiotic or the concentration being used except for strain EC2 with gentamicin at very high concentrations near or above the MIC. This confirms both that the test has a good specificity and that it is not strain-dependent. Note that, apart from the concentration just under the MIC, where the transition occurred, the correct labelling rate (as “No effect” or “ATB effect” according to concentration and strain) was always higher than 97% (with a mean value of 99.3% on the 24 tested conditions excluding the concentration just under the MIC). These results show that the proposed method is not only able to reliably determine the susceptible/resistant phenotype of the bacterial strain at each tested concentration but also to estimate its MIC for each of the tested antibiotics.

## Discussion

In the present work, we addressed the question of performing an AST using Raman spectrometry on single bacterial cells. This technology appeared to be an opportunity to perform a rapid test that would rely on bacterial metabolism analysis instead of cell division. Previous works had shown the ability of Raman spectrometry to discriminate between mechanisms of actions of several antibiotics in *E*. *coli*^21–23^ or vancomycin susceptibility phenotypes in *Enterococci*^23^ but Dekter *et al*. (2016) made the first demonstration of an AST based on an antibiotic-concentration range using Raman spectrometry^22^. The demonstration was performed on a large bacterial biomass previously incubated for five hours in presence of different antibiotic concentrations relevant for a clinical AST test. Here, we addressed this question at the single cell level. We demonstrated that the AST can be performed using Raman spectra acquired on single cells after a two-hour incubation in presence of antibiotic and that only few cells (from 2 × 5 to 2 × 11) were required to perform the test reliably despite an observed intrinsic variability. Thus opening the way to direct sample no-growth analysis, even in pauci-bacterial samples.

Our demonstration first relies on Raman spectra acquisition on single cells and the use of unsupervised classification based on PCA. This first step allowed us to evidence a Raman spectral modification linked to the presence of the antibiotic at inhibitory concentrations. When the bacteria from the susceptible strains were incubated in presence of antibiotic, this signature was expressed with either positive or negative scores leading to two different cell populations (“ATB effect+ ” and “ATB effect−”) co-existing with a third population of cells exhibiting no spectral modifications. When antibiotic concentrations increased, the ratio of these two first “ATB effect+ ” and “ATB effect−” populations increased leading to an overall increased spectral variability of the global population. At antibiotic concentrations above the MIC, modified spectral populations became majority. Spectral heterogeneity has already been described in bacterial colonies^27^ or in a bacterial population that was incubated in the presence of antibiotic^17^. Different publications have also described bacterial phenotypic heterogeneity inside a genetically homogeneous population^28–31^. We excluded that all these cells with non-modified spectra may be “persister cells” such as previously described^32–34^, because the typical occurrence of the “persisters” is in the 10^−4^ to 10^−6^ range, a much lower frequency than the ones we observed. The spectral signature that we evidenced exhibited peaks at 783 cm^−1^, 1240 cm^−1^, 1483 cm^−1^ and 1574 cm^−1^ (with positive weights) and at 1125 cm^−1^ and 1443 cm^−1^ (with negative weights) which were respectively attributed to nucleic acid and proteins by Moritz *et al*.^15^.For these authors, the increase in the intensity of the DNA/RNA peaks was observed during the exponential growth phase, which is also what we observed in a previous work on bacterial micro-colonies grown on agar^25^. Based on our observations, we propose to link the DNA/RNA peaks modification to an apoptosis process. Our hypothesis is inspired from observations on eukaryotic cells, for which the authors described that the 782 cm^−1^ peak increase or decrease corresponded to DNA condensation or DNA fragmentation, respectively^35–37^. Dwyer *et al*.^38^ strengthened this hypothesis by having shown that antibiotic-incubated bacteria exhibit the same hallmarks of DNA/RNA condensation and fragmentation and also related the process to apoptotic process using genetic and proteomic data. Bernatova *et al*. (2013) reported the decrease of the 785 cm^−1^ peak in bacteria incubated with ciprofloxacin and suggested that it could be associated with DNA fragmentation^39^. As a consequence, our hypothesis is that the observed groups with modified spectra – namely the “ATB effect + ” group and the “ATB effect −” group – inside an initially homogeneous susceptible population correspond to groups of cells undergoing DNA condensation and DNA segmentation, respectively. We hence demonstrate that those spectral regions which were previously reported as non-significant due to this high variability by Munchberg *et al*.^16^ in fact contribute on the contrary to the Raman signature of the antibiotic effect and completely explains the associated variability. We could thus evidence the modifications that occurred at the metabolic level of single bacterial cells when they are exposed to antibiotic during two hours. These observations obtained in Raman spectrometry are consistent with other studies realised on larger bacterial biomasses using other methods, showing that antibiotic rapidly affects viability, morphology, some metabolic mechanisms like respiration and related oxidative stress^40–42^, or cell wall synthesis^43^.

After evidencing the Raman spectral signature of the antibiotic effect and the spectral cells heterogeneity, we demonstrated that the AST could be performed despite the intrinsic variability of the acquired spectra and the existence of distinct spectral populations. For that purpose, we were able to train a classifier that allowed us to detect the presence or absence of (spectral) antibiotic effect on a single bacterial cell at a given antibiotic concentration and assign it to one of the classes “ATB effect” and “No effect”. Repeating the test on a small number of exposed bacterial cells (5 to 11 depending on the antibiotic) associated with the same number of non-exposed bacterial cells (to calculate the difference spectra) and making a vote, we could infer the phenotypic antibiotic effect at the scale of the whole bacteria population. Instead of using spectra averaged over a few cells as commonly proposed in the literature, which would result in the destruction of the signal of interest in concentrations close to the MIC, we hence propose to truly analyze bacteria at the single cell level. We leveraged on their heterogeneity for analysis and the proposed vote scheme for the final attribution of the measured phenotype accounts for the observed heterogeneity in bacterial cells state. Using this classification scheme for different antibiotic concentrations, we observed a sharp transition from the “No effect” labelling to the “ATB effect” one at antibiotic concentrations very close to the considered strain reference MIC. We show that the phenotypic reference MIC is also around the concentration, within a standard 2-fold dilution range, at which we could observe a transition of the majority of the cell population from a “no effect” to an “ATB effect+/−” Raman spectrum for susceptible strains. This strongly suggests that we do observe a feature directly linked to the cell capabilities to divide or survive in the presence of antibiotics and that is closely correlated to the observed cell division in a broth micro-dilution test.

If further confirmed on a larger antibiotic and strain panel, in particular concerning the correlation between the observed Raman transition and the reference broth micro-dilution MIC, our method would not only allow one to determine the Susceptible or Resistant phenotype of a given strain but also to estimate its MIC in a very short time since only a few bacterial cells are required in each tested condition to perform the analysis. This would open the way to achieving AST directly from a clinical sample and drastically reduce the time to result. Such strategies would change the paradigm of clinical decision-making from probabilistic initial decision to real-time medicine where initial therapeutic decisions could be based on the results of very rapid phenotype determination.

## Methods

### Materials

*Escherichia coli* strains were obtained from the American Type Culture Collection (ATCC, Manassas, VA, USA) and from bioMérieux Culture Collection (API; bioMérieux, Marcy l’Etoile, France). Culture medium Trypcase Soja (TSA Ref. 43011) and TSB (Ref. 42100) were provided by bioMérieux. Phosphate buffer saline (PBS Ref. A9162, 0100) was purchased from Applichem (64291 Darmstadt, Germany). Gentamicin (Ref. 00768065) was provided by bioMérieux. Ciprofloxacin (Ref. 17850) and amoxicillin (Ref. A8523) were purchased from Fluka-Sigma-Aldrich (Saint Louis, Missouri, USA). Microcon® centrifugal filter (Ref. 42413) device, Microcon® in the text, was from Millipore (Billerica, Massachusetts, USA). Swabs (Ref. 149–0266) were purchased from VWR (Radnor, Pennsylvania, USA). Coverslips (Ref. 0107052) were from Marienfeld (Lauda-Königshofen, Germany). Geneframes (Ref. AB-0577) were purchased from Thermo Scientific (Thermo Fisher Scientific, Inc.). Minimal Inhibiting Concentration (MIC) was controlled either with Vitek® 2 or with e-test®. Bacteria suspension concentrations were measured using a KONTRON UVIKON 940 spectrophotometer. Correspondence between measured optical density at 550 nm and bacteria concentration were calibrated for the tested strains using petri dishes numerations.

### Antibiotic incubation and sample preparation

Efforts were made to minimize variations in sample handling: the two-hour growth and bacterial concentrations were standardized. Strains were stored at −80 °C in broth containing glycerol. Before experiments, a first overnight culture was performed on TSA culture medium at 37 °C. This first culture was stored at 4 °C and constituted a “stock culture” which was used as a single source during the experimental campaign (3 weeks of storage at most). The sample preparation consisted in picking up one colony from this stock culture, diluting in TSB and culturing for 15 h at 37 °C to revitalize the bacteria. For culture, TSB was selected as the preferred liquid culture medium as it is a generic culture medium. The overnight culture was diluted at a final concentration of 5·10^6^ CFU/mL in 5 mL of TSB and followed by a 2-h culture at 37 °C to get bacteria in early stage of growth with less variations between them. The culture was then centrifuged at 1200 × *g* for 8 min. The pellet was suspended in water and was adjusted by dilution to a final concentration of 5·10^7^ CFU/mL. 150 µL of the suspension were diluted in a final volume of 250 µL of PBS containing 10% TSB with varying concentrations of antibiotic and incubated for 2 h at 37 °C. A final 10-fold dilution was selected to express a weaker fluorescence and less pronounced culture medium Raman features on individual bacterial spectra. Bacteria were also prepared without antibiotic using the same protocol. After incubation, samples were then centrifuged at 1200 × *g* for 8 min in the Microcon® device (Millipore), washed with 300 µL of water and centrifuged at 1200 × *g* for 10 min. Bacteria located on the Microcon® device filter were transferred manually from the filter to a coverslip with a swab. Three samples with different antibiotic concentrations and one sample without antibiotic were transferred onto the coverslip. The coverslip was then set upside down onto a geneframe previously sticked on a microscope glass slide.

### Tested antibiotic concentrations

The choice of the tested antibiotic concentrations resulted from a compromise between concentrations used in the reference broth microdilution, the MIC of the tested strains and clinical breakpoints. The objective was to allow the observation of interesting biological phenomena, around the MIC of the tested strain, and to confirm the usability of the method for a Susceptible, Intermediate or Resistant categorization. High concentrations (≥8 MIC) were tested for ciprofloxacin and gentamicin but were not with amoxicillin. Indeed, amoxicillin concentrations superior to 16 µg/ml, caused major filamentations and lysis. These extensive damages to susceptible cells resulted in the impossibility to measure single-cell spectra in such conditions.

### Control culture after antibiotic incubation

Immediately after incubation, an aliquot of each sample was serially diluted in water. 100 µL of dilution were plated onto a TSA culture medium without antibiotic and incubated for 24 h at 37 °C. Bacterial colonies were enumerated to evaluate the concentration of bacteria alive in the different samples. Results are presented in supplementary Fig. 1.

### Raman spectra acquisition

Raman spectra were acquired using a LabRam ARAMIS (HORIBA JOBIN-YVON) equipped with a 600 lines/mm grating, a VENTUS (LASER QUANTUM) 532 nm CW laser and a ZEISS 100x oil immersion objective with a 1.3 numerical aperture. The confocal pinhole was set to 400 µm which results in a measurement volume corresponding to a single cell in this setup. Typical laser power on the sample was measured to be 9 mW and spectra were integrated during 15 s for each single cell. Resulting scattered light was collected on a 1024 × 256 Synapse CCD (HORIBA JOBIN YVON) cooled at −70 °C. To gather spectra, the geneframe holding the coverslip and the samples was loaded on the Raman spectrometer stage. Each spectrum was acquired on a different single cell. A procedure was adopted to decorrelate measures from various potentially confounding factors such as the time delay between sample transfer and spectrum measurement, variation of the substrates or other unplanned experimental condition variations. Five single bacteria spectra were acquired followed by one glass spectrum for a given tested condition. After acquiring those 6 spectra, a new batch of 6 spectra was immediately acquired for a different condition. This process was repeated until all tested conditions of an experiment reached at least 50 gathered spectra.

### Preprocessing of spectra

Each spectrum was composed of 1024 channels corresponding to a Raman shift ranging from 395 to 3075 cm^−1^. Signal measured in spectra mainly originated from fluorescence, in the form of a slowly-varying and intense background with poor specificity, and from Raman scattering of bacteria and glass substrate. As the antibiotic effect of interest was presumably weak, we removed background and glass contribution, with the effect of significantly reducing the impact of non-biological and non-specific spectra variability. Four preprocessing steps were thus used in this study: suppression of “cosmic spikes”, correction of a possible wavenumber shift in the spectra, removal of background and glass contribution, and normalization of spectral intensities by their mean value in the region of interest (ROI) [650–1750] cm^−1^. A more detailed description of the first two and the last steps can be found in a previous publication^25^. Background and glass-contribution removal was done in two steps: we first removed glass contribution (a mean glass spectrum was linearly fitted by lesser values to each bacterium spectrum between 450 and 650 cm^−1^ and subtracted) and then removed the slowly-varying background, mainly due to fluorescence and estimated by peak-clipping using the SNIP algorithm^44^. The resulting spectrum was called the “normalized net spectrum”. Variance values were computed at each wavenumber for a given spectra dataset using the standard definition as the expectation of the squared difference between the intensities and their mean value over the studied dataset, at that given wavenumber. The spectrum obtained by plotting the variance of a dataset for each Raman Shift was called the “variance spectrum” for this dataset, as shown in Fig. 1e and Fig. 2a–c. All preprocessing steps and statistical analyses were carried out in the R software environment^45^.

### Data availability

The datasets generated and/or analyzed during the current study are available from the corresponding author on reasonable request.

## Electronic supplementary material

Supplementary Figures
