# Supplementary material for: Culture-free Antibiotic-susceptibility Determination From Single-bacterium Raman Spectra
Source: Sci Rep. 2018 Mar 2;8:3957. doi: 10.1038/s41598-018-22392-9 (PMC5834538; doi:10.1038/s41598-018-22392-9)
Supplement: Supplementary file 1 — Supplementary Figures [file 41598_2018_22392_MOESM1_ESM.pdf]

# Culture-free antibiotic-susceptibility determination from single-bacterium Raman spectra

A. Novelli Rousseau[a*§], I. Espagnon[b*§], D. Filiputti[a], O. Gal[b], A. Douet[c], F. Mallard[a], Q. Josso[a§]

[a] bioMérieux, Applied Research Department., 5 rue des Berges, 38024 Grenoble, France

[b] CEA, LIST, Département Métrologie, Instrumentation et Information, 91191 Gif-sur-Yvette, France

[c] Bioaster, 40 avenue Tony Garnier, 69007 Lyon, France

*Contributed equally to this work

§ Corresponding authors

## Supplementary Figures

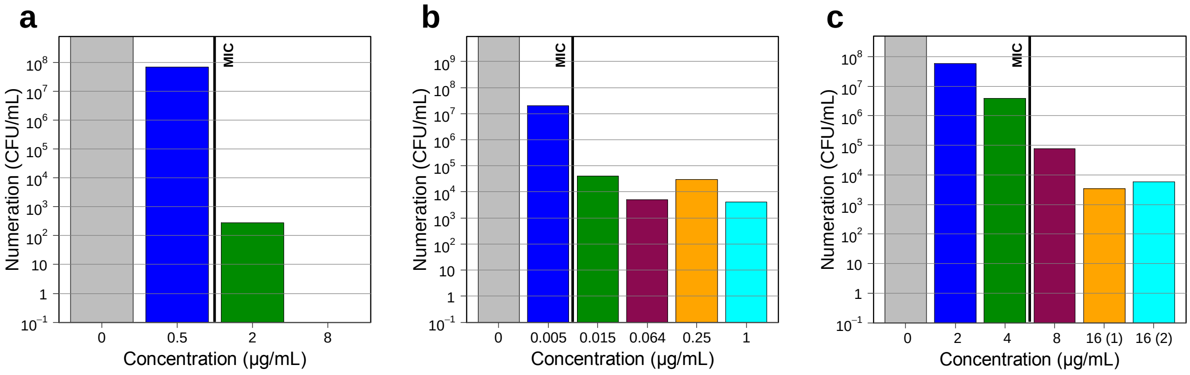


**Supplementary Figure 1. Numerations obtained in viability tests after 2-h incubation** with **(a)** gentamicin, **(b)** ciprofloxacin, **(c)** amoxicillin, at same concentrations as in Fig. 3. Iinitial bacteria concentration before incubation: $3\times10^{7}$ CFU/mL.

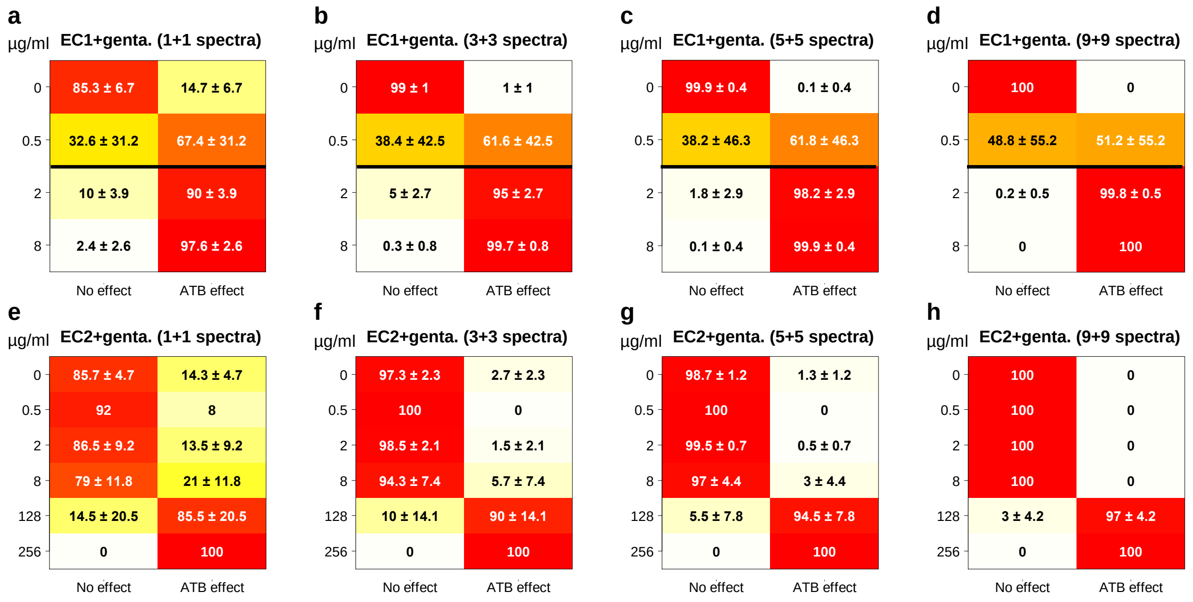


**Supplementary Figure 2.** Confusion matrices for discrimination between classes “No effect” and “ATB effect” obtained at different gentamicin concentrations (**a-d**) for susceptible strain EC1 and (**e-h**) for resistant strain EC2, (**a**, **e**) using 1 spectrum with antibiotic and 1 spectrum without antibiotic – i.e. 1 + 1 spectra –, or (**b, f**) using 3 + 3 spectra, or (**c, g**) using 5 + 5 spectra, or (**d, h**) using 9 + 9 spectra. Black line shows MIC value (1 µg/mL) for susceptible strain. For resistant strain EC2 (**e-h**), the complete transition is observed between 128 and 256 µg/mL.

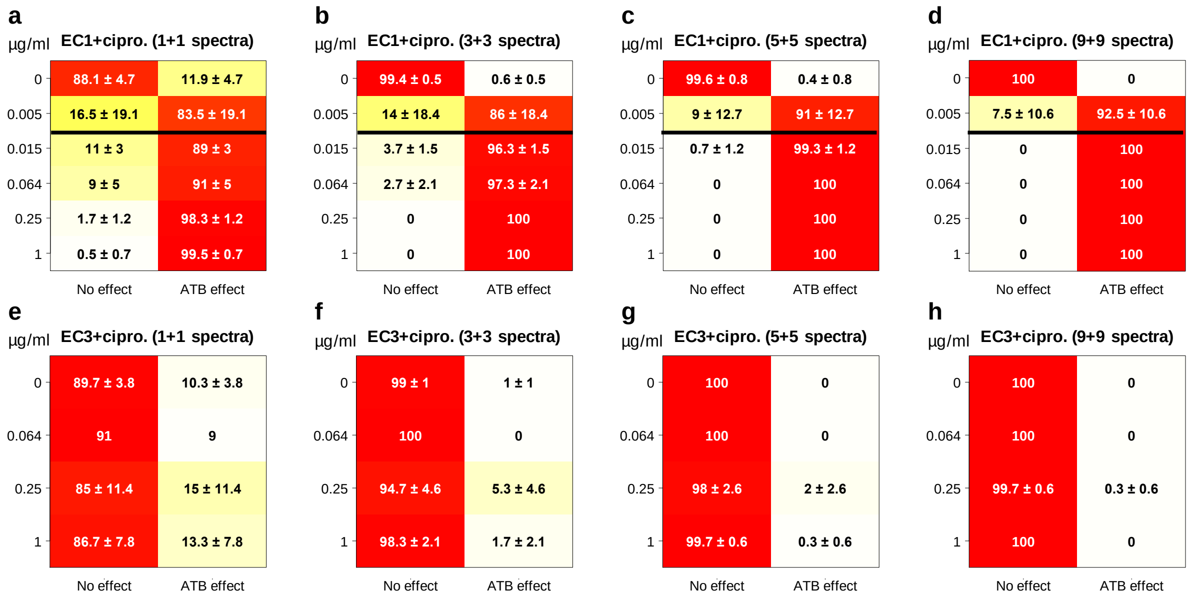


**Supplementary Figure 3.** Confusion matrices for discrimination between classes “No effect” and “ATB effect” obtained at different ciprofloxacin concentrations (**a-d**) for susceptible strain EC1 and (**e-h**) for resistant strain EC3, (**a**, **e**) using 1 spectrum with antibiotic and 1 spectrum without antibiotic – i.e. 1 + 1 spectra –, or (**b, f**) using 3 + 3 spectra, or (**c, g**) using 5 + 5 spectra, or (**d, h**) using 9 + 9 spectra. Black line shows MIC value (0.008 µg/mL) for susceptible strain.

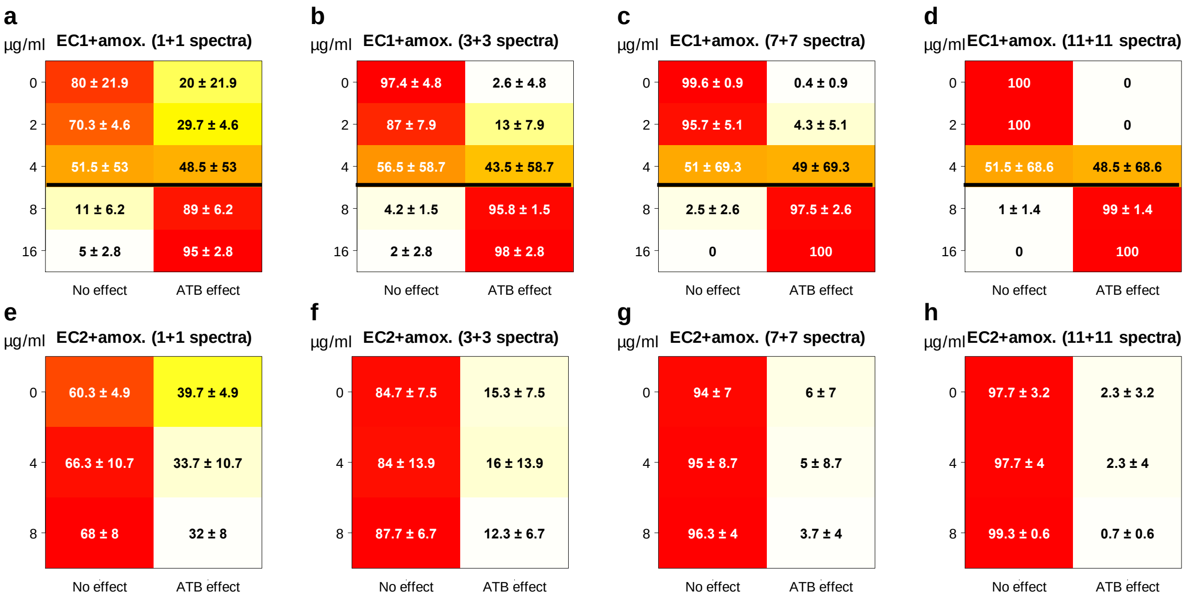


**Supplementary Figure 4.** Confusion matrices for discrimination between classes "No effect" and "ATB effect" obtained at different amoxicillin concentrations (**a-d**) for susceptible strain EC1 and (**e-h**) for resistant strain EC2, (**a**, **e**) using 1 spectrum with antibiotic and 1 spectrum without antibiotic – i.e. 1 + 1 spectra –, or (**b, f**) using 3 + 3 spectra, or (**c, g**) using 7 + 7 spectra, or (**d, h**) using 11 + 11 spectra. Black line shows MIC value (6 µg/mL) for susceptible strain.
